# Lagniappe

**Published:** 1906-08

**Authors:** 


### Lagniappe.

SUGGESTIONS. — Chronic gastritis was treated by the late Dr. William Pepper, with the following pill, which was the great favorite:

℞ Argenti nitratis ..........................
Extracti hyosciami ......................
Vel. pulv. opii ..........................
Mannæ .........................aa gr. iv.

M. et fiat pil No. xii.

---

The following is the best blood tonic I have ever found and has given me some excellent results:

℞ Hydrarg. chlor. corros................gr. iij.

Potass. iodid. ..........................ʒj.

Syr. sarsy. comp.........................

Tr. gentian comp.....................aa ʒiij.

Aqua .......................q. s. ad. ℥ viij.

M. Sig.—Teaspoonful one hour after meals.

J. L. FENNEL, M. D.

---

For inflamed and protruding piles:

℞ Cocain hydrochlorat ................gr. iiss.
Sol. adrenalin. chlorid............1-1000 ʒss.
Bismuth subnit. .......................g. x.
Petrolat ...............................ʒj.

M. et fiat. Unguent.

Sig.—Bathe parts in cold water, and apply ointment with the fingers after each stool. JOHN B. WOODVILLE, M. D.

*—Clipped from Medical Summary.*

Renewing Subscription in advance for the twenty-second consecutive year, Dr. W. J. Collins, The Grove, Texas, says: "May the 'Little Red Rooster' continue to crow for many decades."

---

Jungle Rhymes.—

Mary had a little lamb,
And when she saw it sicken,
She shipped it off to Packingtown,
And now it's labeled "Chicken."

—*Gaillard's.*

Armour had a little cow,
And she had tuberculosis,
He put her up in small tin cans
To sell in broken doses.

Next!

---

Hear Lanphear:

"Full many a gleet of purest germ serene
The dark urethras of the golfers bear;
Full many a maid with blushes all unseen,
Receives the coccus in the open air."

---

Lo, the Poor Editor:

"Lives of great men oft remind us
Honest toil stands little chance;
The more we work we have behind us
Bigger patches on our pants;
On our pants, once new and glossy,
Now are stripes of different hue,
All because our patrons linger
And don't pay us what is due.
Then let us all be up and doing:
Send in your mite, however small;
Or, when the winds of winter strike us
We shall have no pants at all." —*Ex.*

[I have sent out every bill on my books. I need some pants.—Editor.]

---

Some "Phelan" Remarks on a Tender Subject.—The editor of the Oklahoma *News-Journal* remarks:

"Do not make a nitrate of silver apology to a chancroid; caustic remarks are almost as curative, and are furnished gratis by the patient."

And again:

Gabriel—"The last shade is complaining of cramps."

St. Peter—"Tell him we don't treat cholera in phantom."

---

THE LIMIT.—The coinage of words by affixing a Latin or Greek termination to an English stock, as, "lithic acid-emia," for instance, is a barbarism. The latest occurs in the *Medical Council.* Some one there gives a prescription for a "Dandruffcide"! Something to kill dandruff! Soap would be a good dirticide," eh?

---

THE "Red Back" is the journal for *me.* I hope "the game cock of Texas" will continue to crow until we are organized into county, State, and national medical associations, clear of patent medicine men, osteopath, eclectic, homeopath, and negroes.

R. H. SIMPSON.

---

DR. SAM R. BURROUGHS, of Buffalo, in renewing his subscription to the "Red Back" for the twenty-second year, says: "I can not do without it. I have every number from Vol. 1, No. 1, to date."

---

A DISTINCTION of no mean degree has been conferred upon an American book, the joint authorship of Drs. J. Madison Taylor and William H. Wells. The revised second edition of their treatise on "Diseases of Children," published by P. Blakiston's Son & Co., of Philadelphia, has been translated into Italian by Dr. Mario Flamimi, of the Pediatric Clinic of Rome, with contributions by Professor Concetti and Dr. Valagussa. The translation has proven very popular abroad, and the occasion is one of felicitation, not only to the authors, but to American medicine generally, inasmuch as the work was chosen as being especially adapted to clinical teaching in Italy. Few American books have attained such honor. Its success abroad is but a repetition of the favor which it enjoys here.

---

APPRECIATION.

NEW YORK, U. S. A., July 27, 1906.

*Texas Medical Journal, Dr. F. E. Daniel, Austin, Texas.*

DEAR DOCTOR DANIEL: It has just been called to my attention that your journal has reached the twenty-first anniversary of its birth, and I beg you to accept the congratulations of the company upon attaining so dignified an age.

Your publication is so unique that it is read with interest by many men who have no particular interest in the Texas field outside of their regard for the profession at large, and I hope the future of the JOURNAL will be quite as bright as has its past.

Assuring you of our high esteem, I am, with personal regards,

Very truly yours,

H. F. BAKETEL, M. D.,
Advertising Manager Denver Chemical Co.

---

I ENJOY the "Red Back" very much. May success crown your efforts.

Yours fraternally,

W. W. LATHAM.

---

MISSISSPPI VALLEY MEDICAL ASSOCIATION.

OFFICE OF THE SECRETARY,

*Dr. M. M. Smith, Austin, Texas.*

DEAR DOCTOR: The President of the Association has appointed the following delegates to the Congress on Tuberculosis, to be held in New York November 14th to 16th: Drs. Robert H. Babcock, Chicago, Ill.; C. F. McGehan, Aiken, S. C.; Chas. L. Minor, Asheville, N. C., and Wm. Porter, St. Louis, Mo.

Very truly yours,

(Signed) HENRY ENOS TULEY,
Secretary.

